# Use of individual development plans: experiences from junior faculty in the “NURTURE” mentored research program at Makerere University College of Health Sciences

**DOI:** 10.4314/ahs.v22i2.12S

**Published:** 2022-08

**Authors:** Damalie Nakanjako, Grace Banturaki, Harriet Nambooze, Nelson Sewankambo

**Affiliations:** 1 Department of Medicine, School of Medicine, Makerere University College of Health Sciences, P.O. Box 7072, Kampala, Uganda; 2 Infectious Diseases Institute, Makerere University College of Health Sciences, P.O. Box 22418, Kampala

**Keywords:** Individual development plan, career development, academic institutions, LMIC, junior faculty

## Abstract

**Introduction:**

Individual Development Plan (IDP) is a multi-component career planning worksheet that guides trainees through an iterative self-assessment. This paper provides the first investigation of IDP use and experiences among junior faculty at academic institutions in low- and middle-income countries (LMICs) where IDP is seldomly used by trainees.

**Methods:**

An online survey determined the utilization and impact of IDP among junior faculty trainees enrolled on “NURTURE” mentored research program to support career development for faculty at Makerere University College of Health Sciences (MakCHS) between 2016–2020. Responses were received between March and June 2021, a period of intense COVID-19 pandemic in the country.

**Results:**

Of 64 trainees 64(39%) were female and 60/64(98%) developed an IDP during the fellowship period; of whom 45/60(75%) had never been exposed to IDP. Trainees' benefits included intentional thinking about own career goals and support to execute the goals as well as self-management skills of time management and communication, among others.

**Conclusion:**

IDP was well-received by junior faculty trainees, with several self-management and motivation benefits to the scholars. We recommend that academic programs and faculty at academic institutions in LMIC should consider taking on the IDP approach to promote focused career development for all trainees including junior faculty.

## Introduction

In 2002, the U.S. Federation of American Societies for Experimental Biology created the Individual Development Plan (IDP) as a multi-component career planning worksheet that guides doctoral trainees through an iterative self-assessment. IDP provides a platform for trainees to explore opportunities for scientific career growth, aids in the development of short- and long-term career goals, and prompts the creation of action plans to achieve the set career goals^1^. Multiple schools and institutions, both within and without the business field, require their students and/or employees to complete self-assessments and develop professional development plans^2^–^5^. Similarly, NIH created policies that mandate the use of IDPs in NIH-funded training programs. A study of doctoral students in the USA showed that approximately half (53.6%) of the respondents are required to use the IDP while about one third (33.7%) reported that the tool is helpful to their career development^6^.

However, to-date little is known about the use and effectiveness of the IDP tool^6^; particularly in low-and middle-income countries (LMIC) where it is seldomly used by trainees. Most professionals including graduate students and faculty at academic institutions may not intentionally allocate time for self-reflection and introspection on their strengths, weaknesses, as well as career path(s)^7^; which remains a concern for graduate training and faculty development programs at Makerere University College of Health Sciences (MakCHS) and other academic institutions globally. A previous evaluation of doctoral trainees and junior faculty at MakCHS revealed that they needed a structured institutional mentorship program to promote career development and research productivity^8^,^9^. In response to this gap the faculty development team at MakCHS led by Nelson Sewankambo applied competitively and received a faculty development award from the National Institute of Health (NIH), USA, “NURTURE” grant, to provide a mentored research program to support career development, mentorship and research training for junior faculty. Among other support structures to promote development of academic research careers among junior faculty at MakCHS, the “NURTURE” program implemented individual development planning for all faculty trainees that received “NURTURE” research awards. All junior faculty who received competitive “NURTURE” research awards received IDP training at entry and followed through to use of the IDP for the duration of their fellowship period which was at least two years.

This paper provides detailed experiences from the trainees and highlights benefits of using an IDP as perceived by the trainees. To our knowledge this is the first paper to describe qualitative feedback from junior faculty about use of IDP at an academic institution in LMIC The experiences presented here provide encouragement to junior faculty exploring opportunities to develop academic research careers and grow to mentor the next generation of scientists. Obtaining more data on how institutions, faculty and trainees use IDP is predicted to increase its usefulness for exploring both academic and non-academic careers^10^.

## Survey Methodology

**Program setting:** The NURTURE program is a faculty-development program, funded by the National Institutes of Health, USA, designed by senior scientists and trainers at Makerere University College of Health Sciences, and implemented between 2016–2020. The program enrolled junior faculty at the level of senior lecturer and below, who were affiliated to at least one of the departments hosted in one of the four schools of the college of health sciences. During the two-year mentored research fellowship program, each trainee was assigned a team of mentors to support them develop and execute a project to yield peer-reviewed publications in their respective fields of interest. Trainees were also encouraged to apply for grants, and supervise graduate students' research activities; as a strategy to develop their own capacities (i.e., capacities of the NURTURE trainees themselves) and to build the next generation of research leaders in the institution. NIH guidelines for the program excluded sponsoring individuals for degree programs to allow more time for experiential training, although an individual could choose to use his or her experience in the junior mentorship program to contribute to his or her doctoral or postdoc work. A program research administrator coordinated all the trainees' progress activities including but not limited to training on individual development planning and research in progress meetings.

### Survey procedures

The survey was conducted online using the secure web application survey monkey. The survey was distributed to potential respondents through direct email to all the trainees (faculty) who were recipients of the NURTURE mentored-research awards at MakCHS. The emails were followed by phone call reminders to complete the survey and responses were collected over a three-month period, March to June 2021; a period of intense COVID-19 pandemic in the country. The questions in the survey focused on determining the utilization and impact of using an IDP among junior faculty trainees enrolled on the NURTURE program. Quantitative questions included socio-demographic characteristics such as age, gender, duration in university service, number of students supervised at various levels and precoded questions on how using the IDP helped the faculty trainees, how using an IDP may have made life difficult for the trainees, as well as any challenges faced while using an IDP. In addition, the survey included open-ended questions where the faculty trainees were asked to explain each of the responses that were selected. The faculty trainees were also asked to provide any other open-ended responses to the questions provided.

### Data analysis

Quantitative data was analyzed using STATA 12.0 and summarized into frequency tables. Qualitative data was analyzed manually and presented using emerging themes and quotes from the trainees were presented as part of the results. Several quotes were presented verbatim to express the respondents' perspectives. This was part of the NURTURE program monitoring and evaluation plan.

## Results

### Characteristics of trainees who received IDP training during the NURTURE mentored research program at Makerere University College of Health Sciences.

Overall, 64 NURTURE trainees responded to the survey and their demographic characteristics are shown in Table 1. Of those who received IDP training 25/64 (39%) were female and 40% had served in the University service for 10 years and below. Up to 60/64 (98%) of the trainees were able to develop an IDP during the fellowship period, of whom 45/60 (75%) had never been exposed to IDP. (Table 1). At the time of enrollment into the NURTURE program, a majority of the trainees did not have research grants and had not supervised masters or doctoral students (Table 2).

**Table 1:** Demographic characteristics of the faculty trainees at MakCHS that received Individual Development Plan (IDP) training under the NURTURE Program

Variable	Frequency (N=64) %
**Gender**	
Female	25(39.1)
Male	39(60.9)
**Age at time of entry into NURTURE program**	
26 to 35 years	10(15.6)
36 to 55 years	53(82.3)
Above 55 years	1(1.6)
**Duration in University/academic/research service (completed years) at the entry**	
1 to 5 years	22(34.4)
6 to 10 years	18(28.1)
11 to 20 years	23(35.9)
Above 20 years	1(1.6)
**Position of entry into the NURTURE program**	
Assistant Lecturer	12(18.8)
Lecturer	33(51.8)
Senior Lecturer	12(18.8)
Others; specify*	7 (10.8)
**School in the College of Health Sciences that faculty belongs to** λ	
School of Biomedical Sciences	15(23.4)
School of Health Sciences	16(25)
School of Medicine	32(50)
School of Public Health	1(1.6)
**Departments** Ψ	
Clinical departments	54 (84)
Basic science departments	10 (16)
Others; specify.	0
**Prior use of an IDP before NURTURE program**∞	
Never used an IDP	45 (75.0)
Did you develop an IDP during NURTURE program? ∞	
Yes	59(98.3)

Ψ Clinical **Departments included** anesthesia, Child Health and Development Center, Clinical Epidemiology, Dentistry, Family Medicine, Internal Medicine, Lung Institute, Microbiology, Nursing, Obstetrics and Gynecology, Ophthalmology, Pediatrics and Child Health, Pharmacy, Psychiatry and Radiology. **Basic science departments** included Pharmacology, Physiology, Anatomy, Immunology and Clinical Microbiology

* Others included honorary lecturers, research fellows and post-doctoral scientists

λ Each trainee was affiliated to at least one department in one of the four schools at Makerere University College of Health Sciences

**Table 2:** Academic activities of fellows at the time of joining the NURTURE Program

Activity measured	Frequency (%)
**Duration (years) since you joined NURTURE program** *	
5	18 (28.6)
4	23 (36.5)
3	17 (27.0)
2	5 (7.9)
**Number of publications at the time you joined the NURTURE** **program median (IQR)**	8 (4,18)
**Number of masters' students supervised (at the time you joined** **the NURTURE); mean(SD)**	4 (4.2)
**Number of PhD students supervised (at the time you joined the** **NURTURE program) (mean(SD)**	1 (0.5)
**Number of Post-Docs supervised (at the time you joined the** **NURTURE program) mean(SD)**	1 (0.2)
**Fellows who had research grants at the time of joining the NURTURE programme**	
No grant	43 (69.4)
Any research grants	19 (30.7)
**Number of research grants at the time you joined the** **NURTURE program. Median(SD)**	4 (3.6)

* One trainee did not respond to this question

Of the 60 trainees who developed IDP during the NURTURE program, 15 (25%) reported prior use of IDP during pre-doctoral and doctoral training under NIH-funded training programs including the Medical Education for Equitable Services to All Ugandans Consortium (MESAU) (7 trainees), Fogarty Global Health Fellowship (1 trainee), Afya Bora Consortium global health leadership fellowship program (2 trainees), collaborative training program between MakCHS and Case Western Reserve University (CWRU) (1 trainee), workshop under collaborative training program between MakCHS and Yale University (1 trainee), Training Health Researchers into Vocational Excellence (THRiVE) a DELTAS-funded program (2 trainees), and SIDA (1 trainee); see Table 4. Results are presented under two themes: benefits of using an IDP and how use of IDP helped the trainees; as summarized below

**Table 4:** Experiences of junior faculty with Individual Development plans

**Theme**	**Responses from NURTURE trainees**

**Prior use of IDP**	45 out of 60 respondents (75%) had never been exposed to IDP

	15 out of 60 respondents (25%) reported prior use of IDP during pre-doctoral and doctoral training During doctoral training under MEPI-MESAU (7) During doctoral training under THRiVE (2) During doctoral training under SIDA (1) Workshop under collaborative training program between MakCHS and YALE University (1) During training collaborative training program between MakCHS and CWRU (1) During Fogarty Global Health Fellowship (1) During Afya Bora Consortium global health leadership fellowship program (1)

**How use of IDP helped (from all the trainees who used it during the NURTURE** **Program)**	

	Goal setting Keeping focus on the set goals Monitoring progress and accomplishments Self-reflection Change of strategy in order to meet set goals Motivation for career development Looking out for opportunities and networking Time management Other skills; communication, self-awareness, attitude change

**How use of IDP made life difficult**	

	Setting my pace Handling failure

IDP, Individual development plan, Medical Education for Equitable Services to All Ugandans Consortium (MESAU), Training Health Researchers into Vocational Excellence (THRiVE), Swedish International Development Agency, MakCHS Makerere University College of Health Sciences, Kampala, Uganda, CWRU Case Western Reserve University, Cleveland, USA. Further details are provided in the quotes presented in the results section.

**Benefits of using an IDP:** Trainees experienced several benefits of developing and using an IDP including being forced to intentionally think about career goals and receive support to execute the goals as well as some self-management skills such as time management and communication skills (Table 3). Up to 41/58 (71%) of the trainees made modifications of the IDP as appropriate during the fellowship period and 33/67(49%) received promotions during the fellowship period. Only 4/40 (10%) of the trainees reported continued use of the IDP after their NURTURE fellowships ended (Table 3).

**Table 3:** Benefits and challenges of using an IDP as reported by NURTURE program fellows

Fellow assessment reportΨ	Yes	No
**Benefits of using IDP** *		
It helped/forced me to think much more about my goals as a faculty member	57(95)	3(5.0)
I dedicated time to reflect on my goals	56(96.5)	2(3.4)
I was able to monitor my career progress.	55(94.8)	3(5.2)
I have been able to support other colleagues to develop an IDP	38(62.5)	20(34.5)
I have been able to support my students to develop an IDP	35(60.3)	23(39.7)
I improved my time management skills	50(86.2)	8(13.8)
I improved communication with my mentor/supervisor	46(79.3)	12(207)
Did you have to modify/update your IDP at any time during the NURTURE fellowship	41(70.7)	17(29.3)
Since you completed the NURTURE program, do you still use your IDP?	4(10.0)	36(90.0)
Have you received any promotion or addition of academic responsibilities since you started using an IDP to monitor your career progression	33(57.9)	24(42.1)
**Challenges of using IDP**		
IDP made my life difficult	5(8.6)	53(91.4)
It was difficult for me to monitor my IDP	46(79.3)	12(20.7)
I still need more help on how to develop an IDP.	21(36.2)	37(63.8)

Ψ Multiple responses per individual were acceptable. Fellows selected only the responses that were applicable.

* Four trainees did not respond to this question

The details of how these benefits were attained are explained in the qualitative data which is summarized below and in Table 4.

How use of IDP helped, as reported by the NURTURE trainees, is presented below in sub-themes that emerged from the respondents;

**a) Goal setting:** Use of IDP helped trainees to set their respective career development goals as quoted below

*“I was able to set my goals for the future including the papers and grants I wanted to write”, repondent 1*

*“The IDP is structured so it helped me develop my objectives precisely and clearly” repondent 2*

*“I drew up research and career development goals with measurable outputs and timelines which made it easy to track”* respondent 3

*“I was able to clearly state where I want to be in the next 5 years”* respondent 4

**b) Keeping focus on the set goals:** Use of IDP helped trainees to keep focused on their respective goals, as quoted below.

*I became focused on achieving my targets the limitations not withstanding”*

*“The IDP constantly reminded me of what I needed to do within a specified period of time”*

*“I made focused consultations”,* said the trainees.

**c) Monitoring progress and accomplishments:** Use of IDP helped trainees to monitor their own progress.

*“I would frequently examine my IDP for progress made and I would try to work on the challenges encountered during that period”.*

*“I regularly (usually once a month) check my IDP to ensure that I remain focused on my goals as faculty”*

*“At the beginning of my fellowship I set goals (with my mentors and supervisors), I worked on them during the fellowship with specific activities, and I achieve majority of the goals I set”*

*“I can now monitor my annual progress”*

*“I was able to quantify my goals and successes in terms of numbers which made this a reality”,* said the trainees.”

**d) Self-reflection:** Use of an IPD helped trainees to dedicate time to reflect on their respective careers, as illustrated in the quotes below.

*“I was able to think coherently how to move along a career path”*

*“As I reflected on my goals, I was able to evaluate my progress objectively and I was able to tick off all planned activities”*

*“I usually took off two hours a month to reflect on my goals and check if they are still relevant and realistic”*

*“In my IDP I have dedicated two hours every week to reflect on my goals”*

*“Whenever I thought of a goal, I also had to think of a method of achieving it”*

*“Before I started using an IDP, I would wake up and do whatever came my way, but now I plan my day and give priority to my set goals”*

*“An IDP is a journey,”* said the trainees.

**e) Change of strategy in order to meet set goals:** Use of an IDP helped trainees to make relevant changes in their career progression processes, as illustrated in the quotes from the trainees below.

*It helped me to search, collaborate and purpose my training towards building my research and publication capacity”*

*“I started being intentional about my choices, and I sought a scholarly mentor”*

*“It helped me strategize on the steps I would take to achieve my goals by breaking them into small achievable steps”*

*‘I identified that I needed to change to a faster analysis platform to handle the volume of work I had ahead and within 6 months I had changed from STAT A to R for data analysis”*

*“I was able to review goals that needed to be revised and updated”*

*“IDP helped me to identify and improve on areas where I have been weak, like supervision as well as my research inputs and outputs”*

*“I was able to turn down opportunities that were not towards my goals since they were distractors,”* said the trainees.

**f) Motivation for career development:** Use of an IDP helped the trainees to remain self-driven to achieve their respective career goals, as illustrated by the quotes below.

“*I printed my IDP and pinned it in my office. It has been and continues to be a reminder of what I need to accomplish”*

*“As a faculty, it motivated me to focus on resource mobilization through grant writing”*

*“I learned that my career progression depended on my scholarly outputs so I became more conscious about them through my IDP”*

*“My IDP was like my career development tracker”*

*“It was a guiding document that simplified my life”*

**g) Looking out for opportunities and networking:**

Use of an IDP helped trainees to network and harness opportunities for collaborative training and research activities, as quoted from the trainees below;

*“By referring to my objectives as written in the IDP, I was forced to look/shop for opportunities, and ultimately meet my goals”*

*“I was forced to look for grant opportunities, and collaborative networks abroad and planned to start a PhD”*

*“It helped me focus and be intentional to create a network of researchers for collaboration”*

*“I poke to my mentor about my interest in pursuing a PhD and he linked me to potential supervisors in my field”*

*“I was encouraged to attend “research in progress” meetings with other researchers in order to learn from other people and build my skills and network”*

**Time management:** Use of an IDP enabled trainees to keep track of their time and modify time commitments in order to achieve their respective goals. This is illustrated in the quotes below.

*“It kept me in check regarding accomplishment of my goals on time”*

*“I improved my time management and time allocation”*

*“I gave a realistic timeframe to each of my goals”*

*“I fixed extra time. It had been difficult to achieve because of busy schedules”*

*“Because I had timelines, I had to reflect on how to improve whenever I was behind schedule”*

*“I have pinned a copy of my IDP on a wall in my house to remind myself of the timelines”*

*“I always have a component of my IDP on my to-do-list”*

*“I apportioned my work time as follows; 50% on teaching and supervision of students, 40 % on grant application and 5% on community activities”*

*” I set for myself an academic day each week”*

*“At the moment I do not pile up appointments I will not attend and I try to finish an assignment 2 days before the due date”*

h) Mentoring: Use of an IDP helped trainees to develop interest in mentoring other people, as illustrated by the quotes from the trainees below.

*“I was able to interest postgraduate students that I have mentored to do PhD as I mentor them”*

*“I improved the way I teach and supervise students' research work”*

*“My mentoring has greatly improved, cumulatively I have mentored over 10 trainees at different levels”*

*“I am currently mentoring a student in a program that requires the student to make an IDP. The fact that I already had to develop one myself makes my mentoring easy and I continue to improve”*

*“I was able to share my IDP with research fellows in my institute, discuss with them the benefits and share the template in one of the capacity building sessions”*

i) Other skills including communication, self-awareness and resilience: Through using an IDP fellows developed other soft skills that were required for their success, as illustrated in the quotes from the trainees below.

*“I learned to celebrate each milestone and inspire myself to move on”*

*“After failing to achieve planned goals I sit and redesign my approach”*

*“My experience with the IDP helped me organize other aspects of my life- I used a similar approach to my social life”*

*“Balancing lifestyle with work became much easier for me”*

*“I interact better with people around me because I am able to categorize issues and assess their impact on my goals”*

*“Using an IDP helped me enforce good work ethics”*

*“I learned to make goal-oriented communications”*

*“I improved my listening skills”*

*“Using an IDP helped me to identify my gaps and utilize feedback”,* said the trainees.

How use of an IDP made life difficult for the trainees. Fellows explained that using an IDP made their lives difficult in a rather productive way, as described in the quotes below.

*“It made my life difficult but in a good way. It helped to push me beyond my usual limits and it kept me on my toes”*

*“The IDP drives you”, “I still cannot believe everything that I achieved in a short time”*

*“IDP made me create a new normal in my career development activities”*

*“The thought of having to shift timelines when the goals are not achieved was haunting”*

*“The reverse is true; my life is a lot easier with an IDP which helped to cancel some time consuming “beneficial” activities so that I remain focused on my goals”*

*“The IDP involves several contributors who are not in my control”*

*‘It was not difficult because everything was well documented and easy to track,”* said the trainees.

Additional help needed with the IDP. Fellows reported other personal benefits from the learning of using an IDP, as quoted from the trainees below.

*“I need support on how to tie the loose ends together, more so when the expectations are not met according to the stipulated timelines”*

*“I need more help on how to set attainable goals”*

*“I need more help on how to effectively monitor my IDP”*

*“I need more help on how to identify suitable mentors”*

*“I want to see how other people tracked their IDPs so that I learn from their experiences”*

*“I need more help so that I can teach all my mentees on how to use an IDP”,* said the trainees.

## Discussion

In a survey to evaluate use of IDP among junior faculty on a mentored research fellowship, we found that up to 98% of the trainees were able to develop an IDP during the fellowship period; despite the fact that a majority (75%) had never been exposed to IDP. We reported high use of the IDP among trainees who were introduced to the tool at the beginning of their mentored research fellowship. This is a lot higher than previous reports in USA among postdoctoral researchers (19% used IDP) and their mentors (9% used IDP), although the perceived value of the instrument was high for those who had used the tool; 71% for postdoctoral researchers and 90% for mentors respectively^1^. Trainees had a wide spectrum of benefits of using an IDP with a detailed account of the processes and trainees' experiences. Fellows reported numerous benefits from using an IDP which included among others ability to set career goals, keeping the focus to achieve the set goals, reflection on individual accomplishments, strategizing for unmet goals, networking and ability to mentor other mentees on use of IDP. Our results are encouraging that most trainees took to heart IDP and were influenced positively to work hard and focus on their respective career development goals.

This report provides answers to most of the frequently asked questions about how trainees implement the IDP and how an IDP modifies the activities and priorities of the learner/faculty member/scientist to set them up for success. Career development support and related infrastructure for doctoral trainees had been suggested as a critical element to sustainability of the biomedical workforce^11^. A previous survey among doctoral students in the USA showed that respondents who were confident about their career plans and who took advantage of career development resources at their institution were more likely to perceive that the IDP is useful for their career development^6^. Given the benefits of IDP presented by faculty trainees in health professions at MakCHS and among multiple schools and institutions both within and without the business fields and higher education^2^, ^4^, ^5^, academic institutions in LMIC need to adopt the IDP approach to promote career development and productivity in all disciplines.

Despite the many benefits of the IDP during the NUR-TURE fellowship program, only 10% of the trainees reported using the IDP beyond the fellowship program. It is likely that trainees had completed the career goals set for the two-year fellowship period and needed to set new goals. Similarly, only a few passed on the IDP experience to their mentees. The qualitative responses showed that trainees needed more support to be able to pass on their IDP experiences to their respective mentors/supervisors and mentees/supervisees. This is likely because there is no formal policy on integration of IDP in curricula of formal academic programs although some mentors use it informally to support their mentees/supervisees. Some trainees in this report mentioned the need for more training and support to adopt the IDP strategy to routine mentoring processes for students and faculty. We therefore recommend that academic programs and faculty in all colleges should consider taking on the IDP approach to promote focused care development for all trainees including junior faculty. Further work is needed to document and deal with any challenges to development of a culture of using IDP to support academic career development in LMIC.

## Limitations

This study is limited to experiences of the individuals using the IDP (from the individual's perspective) and does not explore experiences and lessons learned by the program directors or institutional leadership. However, it is evident, from our results that using an IDP promoted a great deal of introspection, reflection and self-awareness among the users, which is a proxy for career growth. Prior research indicates a positive relationship between self-awareness and overall leadership success^12^, ^13^. Similarly, the lack of self-awareness has been related to the failure to meet objectives, which is a key predictor of leadership failure in addition to inability to adapt to transitions and changes, interpersonal relationship problems and team leadership breakdown^7^. Awareness of one's own leadership strengths and weaknesses, as well as a strategic understanding of the path(s) to follow to achieve one's future professional goals, are both required to design short-term and long-term career goals^7^. Despite this study's limitations, this is the first investigation of IDP use and experiences among junior faculty at academic institutions in LMICs. Thus, this work provides a baseline understanding of the use of IDP in this population and it should promote additional research on the approach, and strategies to scale up this intervention to promote career development.

## Conclusion

Individual development planning was well-received by junior faculty/fellows in a mentored research program at Makerere University College of Health Sciences, with several self-management and motivational benefits to the scholars. There is need to sustain career development support to grow successful leaders and mentors for the next generation of academic leaders. We recommend that academic programs and faculty at academic institutions in LMIC should consider taking on the IDP approach to promote focused care development for all trainees including junior faculty.
